# Hepatitis E should be considered a neglected tropical disease

**DOI:** 10.1371/journal.pntd.0007453

**Published:** 2019-07-25

**Authors:** Andrew S. Azman, Iza Ciglenecki, Joseph F. Wamala, Julia Lynch, Rakesh Aggarwal, Mahmudur Rahman, Sid Wong, Micaela Serafini, Ali M. Moussa, Harry R. Dalton, Ananta Shrestha, Rajendra Pant, Raquel Peck, Emily S. Gurley

**Affiliations:** 1 Department of Epidemiology, Johns Hopkins Bloomberg School of Public Health, Baltimore, Maryland, United States of America; 2 Médecins Sans Frontières, Geneva, Switzerland; 3 World Health Organization, Juba, Republic of South Sudan; 4 International Vaccine Institute, Seoul, South Korea; 5 Department of Gastroenterology, Sanjay Gandhi Postgraduate Institute of Medical Sciences, Lucknow, India; 6 Infectious Disease Division, icddr,b, Dhaka, Bangladesh; 7 Medical Department, Médecins Sans Frontières, Amsterdam, the Netherlands; 8 Chad Ministry of Health, Department of Communicable Diseases, N’Djamena, Chad; 9 Truro, Cornwall, United Kingdom; 10 Liver Foundation Nepal, Kathmandu, Nepal; 11 SAARC TB and HIV/AIDS Centre, Kathmandu, Nepal; 12 World Hepatitis Alliance, London, United Kingdom; Lowell General Hospital, UNITED STATES

Hepatitis E virus (HEV) is a major cause of acute jaundice worldwide, with a case fatality risk (CFR) as high as 30% among symptomatic pregnant women. Human HEV includes four genotypes; the nonepidemic, zoonotic genotypes (typically 3 and 4) have gained increasing recognition in North America and Europe over the past decade [1] because of the potential risk of transmission through food, blood transfusions, and organ donation. However, relatively little attention and few resources have been invested into genotypes 1 and 2 (g1/g2), which cause outbreaks among the world’s most vulnerable populations living with poor access to safe water and sanitation infrastructure. Here, we argue that the burden of this disease, combined with the neglect by the public health, research, and clinical communities and limited options for treatment and control, make it a serious candidate for classification as a neglected tropical disease (NTD).

HEV g1/g2 are transmitted through the fecal–oral route, so large water and sanitation interventions have been implemented during outbreaks to reduce transmission. The few studies that have looked into intervention impact during outbreaks provide little evidence of major intervention-related reductions in risk, especially during outbreaks in Africa [2,3]. However, studying the effects of these interventions is difficult because of the long incubation period of the disease and relatively imprecise diagnostics and clinical case definitions [2–4]. There are no therapeutics known to reduce morbidity and mortality from acute hepatitis E in the general population, although antivirals may provide some hope in the future [5]. Fortunately, there is a vaccine licensed for use in China, Hecolin (Xiamen Innovax Biotech, China), that has demonstrated high efficacy in a large randomized trial [6]. However, this vaccine is not approved for purchase by United Nations organizations (World Health Organization [WHO] prequalified), nor has it been used in routine public health interventions.

The classification as an NTD by WHO can bring additional dedicated public health and research resources to a disease, in addition to increasing general awareness in the health community and beyond. Recent additions to this list include chromoblastomycosis and other deep mycoses, scabies and other ectoparasites, and snake bite envenoming [7]. The WHO Strategic and Technical Advisory Group for Neglected Tropical Diseases currently lists 20 recognized NTDs and has set out the criteria that should be met for future additions to the list [6]. These criteria are as follows: (1) the condition disproportionately affects populations living in poverty and causes important morbidity and mortality in such populations, justifying a global response; (2) the condition primarily affects populations living in tropical and subtropical areas; (3) the condition is immediately amenable to broad control, elimination, or eradication by applying one or more of the five public health strategies adopted by the (WHO) Department for Control of NTDs; and/or (4) the condition is relatively neglected by research when it comes to developing new diagnostics, medicines, and other control tools. Here, we review these four criteria with respect to hepatitis E caused by epidemic HEV (primarily g1/g2) and provide the evidence to support its inclusion as an NTD.

## Criterion 1: The condition disproportionately affects populations living in poverty and causes important morbidity and mortality in such populations, justifying a global response

Outbreaks of HEV g1/g2 are almost exclusively reported from countries with a low to medium human development index in Africa and Asia, with an estimated 50,000 HEV-related deaths occurring annually, mostly attributable to g1/g2 [8,9]. Vulnerable subpopulations within these countries are frequently affected, including displaced persons [2,10,11] and those living in urban slums, where water and sanitation services and health infrastructure are poor [12–14]. Pregnant women, another vulnerable subpopulation, are among those most likely to die from symptomatic disease, with a CFR of up to 30% [13,15]. Although there is limited evidence to assess the relative risk of HEV infection or mortality among persons living with HIV compared with the general population, those with advanced liver disease are known to be at increased risk for severe outcomes from HEV infections [16].

Travel-related hepatitis E (g1/g2) cases are reported in Europe and North America, although there is little evidence of onward transmission from those cases, suggestive of the critical role of water and sanitation infrastructure in reducing risk [4,17,18]. Given that HEV tends to circulate in areas where surveillance systems are weak, that the clinical case definition of hepatitis E is nonspecific, and the high proportion of mild or asymptomatic infections [8,9], global estimates of hepatitis E incidence (infection and/or disease) and mortality are poor. Our current understanding of the burden of hepatitis E g1/g2 come from three primary sources: (1) surveillance or studies related to “outbreaks,” (2) routine surveillance data from hospitals, and (3) serological studies. Two recent systematic reviews of outbreaks of HEV highlighted that outbreak size and CFR vary considerably between settings, with some outbreaks reporting tens of thousands of clinical cases and CFRs ranging from 1%–3% among the general population and from 5%–30% among pregnant women [13,15]. Even in settings with no reported hepatitis E outbreaks, seroprevalence studies have suggested the presence of ongoing transmission, with many older serologic surveys likely underestimating true seroprevalence because of insensitive diagnostics [15,19,20]. Studies in Bangladesh estimated that 9%–25% of maternal deaths are associated with acute jaundice, with hepatitis E as the likely cause of the majority of these deaths [21–23].

## Criterion 2: The condition primarily affects populations living in tropical and subtropical areas

Reports of hepatitis E come primarily from tropical and subtropical areas, including the parts of Africa and Asia described previously [13]. Despite decades of global efforts, 30% of the world’s population still lives without consistent and adequate access to safely managed drinking water [24], and the contamination of their water with human feces puts them at risk for HEV infection. These people predominantly live in the world’s poorest countries in tropical and subtropical areas, where surveillance systems to aid in the control of outbreaks are often inadequate.

## Criterion 3: The condition is immediately amenable to broad control, elimination, or eradication by applying one or more of the five public health strategies adopted by the Department for Control of NTDs

The best way to prevent HEV g1/g2 transmission is through universal access to (and use of) safely managed water and sanitation, as evidenced by the lack of outbreaks in high-income settings. The populations most at risk for hepatitis E g1/g2 include some of the hardest to access, where sustainable gains in water and sanitation may be decades away. Fortunately, vaccination may provide a unique opportunity to reduce the burden of disease on both individuals and health systems. Whereas other genotypes of hepatitis E have known zoonotic reservoirs [25], g1/g2 do not, making these genotypes more plausible candidates for elimination through public health interventions.

## Criterion 4: The condition is relatively neglected by research—i.e., resource allocation is not commensurate with the magnitude of the problem—when it comes to developing new diagnostics, medicines, and other control tools

Hepatitis E research is neglected by traditional research funders. We conducted a nonexhaustive search of recently awarded grants from three large biomedical research donors and found very few investments in hepatitis E. Although there is no perfect comparator disease, we found that compared with cholera and typhoid, two diseases that are often considered neglected, transmitted through similar pathways, and vaccine preventable, investments in HEV are relatively small (Table 1). For example, the United States National Institutes of Health invested roughly 0.6 million USD in hepatitis E research per year from 2008 to 2017, whereas during the same time period, they invested 17.3 million USD per year in cholera and 8.9 million USD per year in typhoid-related research.

**Table 1:** Overview of grants awarded with “hepatitis E,” “cholera,” or “typhoid” in the grant title or description by major donors in the United States of America and United Kingdom. Note that this is an illustrative example, and we were unable to identify other open databases of investments for other relevant donors.

Donor	Average Annual Investment in Hepatitis E (USD)	Average Annual Investment in Cholera (USD)	Average Annual Investment in Typhoid (USD)	Time Period	Source
US National Institutes of Health	$590,000	$17,390,000	$8,969,000	2008–2017	https://federalreporter.nih.gov/projects/search/
The Bill and Melinda Gates Foundation	$64,000	$6,608,000	$13,573,000	2010–2017	https://www.gatesfoundation.org/How-We-Work/Quick-Links/Grants-Database
Wellcome Trust*	$0	$432,000	$1,085,000	2005–2017	https://wellcome.ac.uk/funding/grants-awarded

*Conversion used is 1 pound = 1.27 USD.

Only one hepatitis E vaccine, a genotype 1 recombinant vaccine, Hecolin (Xiamen, China), has been tested in a phase III trial and was found to be safe and effective against sporadic hepatitis E genotype 4. This demonstrated cross protection, and the fact that all known genotypes belong to a single serogroup suggests the vaccine will protect across genotypes [26]. However, because of the lack of clear recommendations for when to use the vaccine [27], the lack of WHO prequalification, and simply the lack of experience with using this vaccine, it has never been adopted within a national public health system. Hecolin is currently licensed for use among nonpregnant persons aged ≥16 years because of a lack of evidence in other groups, limiting the potential impact on mortality and morbidity at the individual- and population-level. Modeling results suggest that reactive vaccination against hepatitis E outbreaks may help avert significant numbers of deaths [28]; however, this has not been tested in practice and remains an important research question. We believe that well-documented experiences with this vaccine in epidemic and endemic settings will help inform more specific global policy recommendations and help countries feel more confident about adopting the use of the vaccine.

## Conclusions

Hepatitis E disease caused by HEV g1/g2 has a significant burden globally, especially among the most vulnerable subtropical populations. The disease is amenable to broad control with improvements in infrastructure and vaccination programs and should be classified as an NTD. Even if the current vaccine obtains WHO prequalification, widespread use and increased investment (e.g., by GAVI, the vaccine alliance) will likely remain limited until we have better data on disease burden and disease risk and evidence to support use of the vaccine in expanded populations and settings as noted by WHO [26]. If prevention tools are readily available, then the resources for surveillance are easier to justify. If burden-of-disease data highlight key areas with elevated incidence and/or mortality, they can spur development and deployment of cost-effective interventions. As with many other NTDs, the historically limited investments in hepatitis E g1/g2 have led to this vicious cycle, in which limited tools and knowledge preclude new investments. Advances in reducing the burden of epidemic hepatitis E may be extremely difficult without investments in both defining the problem and identifying new solutions, across sectors.

Classification as an NTD alone will certainly not alleviate the suffering from this disease in the short term, but it may help break the cycle of neglect by accelerating advances in research and public health action and elevating epidemic hepatitis E in the global health agenda. Although we focused specifically on g1/g2, this level of specificity may make it harder to communicate, and there may be benefits for focusing on all genotypes of hepatitis E, especially because we know little about the burden and distribution of genotypes 3 and 4 in lower-income countries. With the recent release of “Recommendations to assure the quality, safety and efficacy of recombinant hepatitis E vaccines” (http://www.who.int/biologicals/expert_committee/POST_ECBS_2018_Recommendations_HEP_E_vaccines.pdf?ua=1), a clear pathway for prequalification of hepatitis E vaccines exists, and we urge rapid progress so that this valuable tool can easily be used by countries experiencing hepatitis E outbreaks. We also urge donors to invest in research ranging from basic science to studies of transmission to the development of interventions and testing of effective intervention strategies. Finally, we advocate for discussions within WHO and with countries affected by HEV g1/g2 to further develop the case for classification as an NTD.
